# Prognostic model for overall survival that includes the combination of platelet count and neutrophil–lymphocyte ratio within the first six weeks of sunitinib treatment for metastatic renal cell carcinoma

**DOI:** 10.1186/s12885-022-10316-w

**Published:** 2022-11-24

**Authors:** Miki Takenaka Sato, Ayuki Ida, Yuki Kanda, Kaori Takano, Masayuki Ohbayashi, Noriko Kohyama, Jun Morita, Kohzo Fuji, Haruaki Sasaki, Yoshio Ogawa, Mari Kogo

**Affiliations:** 1 Division of Pharmacotherapeutics, Department of Clinical Pharmacy, Showa University School of Pharmacy, 1-5-8 Hatanodai, Shinagawa-ku, Tokyo, 142-8555 Japan; 2 Department of Urology, Showa University School of Medicine, Tokyo, Japan; 3 Department of Urology, Showa University Northern Yokohama Hospital, Yokohama, Japan; 4 Department of Urology, Showa University Fujigaoka Hospital, Yokohama, Japan

**Keywords:** Sunitinib, Renal cell carcinoma, Combination of platelet count and neutrophil–lymphocyte ratio (COP-NLR), Prognostic factor, Prognostic model

## Abstract

**Background:**

The association between the combination of platelet count and neutrophil–lymphocyte ratio (COP-NLR) at the time of adverse events during sunitinib treatment and prognosis is unclear, and prognostic models combining the prognostic factors of sunitinib have not been well studied. Thus, we developed a prognostic model that includes the COP-NLR to predict the prognosis of patients with metastatic renal cell carcinoma (mRCC) treated with sunitinib.

**Methods:**

We performed a retrospective cohort study of 102 patients treated with sunitinib for mRCC between 2008 and 2020 in three hospitals associated with Showa University, Japan. The primary outcome was overall survival (OS). The collected data included baseline patient characteristics, adverse events, laboratory values, and COP-NLR scores within the first 6 weeks of sunitinib treatment. Prognostic factors of OS were analyzed using the Cox proportional hazards model. The integer score was derived from the beta-coefficient (β) of these factors and was divided into three groups. The survival curves were visualized using the Kaplan–Meier method and estimated using a log-rank test.

**Results:**

The median OS was 32.3 months. Multivariable analysis showed that the number of metastatic sites, Memorial Sloan Kettering Cancer Center risk group, number of metastases, non-hypertension, modified Glasgow Prognostic Score, and 6-week COP-NLR were significantly associated with OS. A higher 6-week COP-NLR was significantly associated with a shorter OS (*p* < 0.001). The β values of the five factors for OS were scored (non-hypertension, mGPS, and 6-week COP-NLR = 1 point; number of metastatic sites = 2 points; MSKCC risk group = 3 points) and patients divided into three groups (≤ 1, 2–3, and ≥ 4). The low-risk (≤ 1) group had significantly longer OS than the high-risk (≥ 4) group (median OS: 99.0 vs. 6.2 months, *p* < 0.001).

**Conclusions:**

This study showed that the COP-NLR within the first 6 weeks of sunitinib treatment had a greater impact on OS than the COP-NLR at the start of sunitinib treatment. The developed prognostic model for OS, including the 6-week COP-NLR, will be useful in decision-making to continue sunitinib in the early treatment stage of patients with mRCC.

**Supplementary Information:**

The online version contains supplementary material available at 10.1186/s12885-022-10316-w.

## Background

Renal cell carcinoma (RCC) accounts for 5% of all cancers in men and 3% of cancers in women worldwide, representing the 6th and 10th most frequently diagnosed cancers, respectively [1, 2]. The 5-year survival rate is 74% overall, decreasing to 8% among patients with metastatic disease (stage IV) [3, 4]. Early-stage RCC is often asymptomatic, although the presence of systemic symptoms is frequently associated with advanced or metastatic RCC (mRCC).

The treatment selection for patients with mRCC widely uses the Memorial Sloan Kettering Cancer Center (MSKCC) and International Metastatic Renal Cell Carcinoma Database Consortium model. The advent of immune checkpoint inhibitors has broadened the treatment options for mRCC. Although sunitinib is one of the first-line treatment regimens for patients with low-risk mRCC, it must be discontinued if it causes severe bone marrow suppression. However, it has been reported that long-term treatment with sunitinib at a dose that reduces tumor size in the early stage is critical to maximize the potential efficacy of sunitinib treatment [5]. Therefore, determining the clinical benefit of continuing sunitinib prior to the occurrence of serious adverse events (AEs) leads to an appropriate treatment option for mRCC.

Previous studies have reported that C-reactive protein (CRP), MSKCC model, modified Glasgow Prognostic Score (mGPS), and malnutrition are significantly associated with prognostic factors for progression-free survival (PFS) and overall survival (OS) of mRCC treated with sunitinib [6–11]. Additionally, sunitinib-induced hypertension, neutropenia, and thrombocytopenia have been reported as predictors of sunitinib efficacy [12–15]. Moreover, one study has developed prognostic models by combining the prognostic factors of sunitinib [7].

On the other hand, the combination of platelet count and neutrophil–lymphocyte ratio (COP-NLR), which is calculated using inflammatory markers, such as the NLR and platelet count (PLT), has been shown to be useful as a prognostic factor in gastrointestinal cancer and non-small cell lung cancer [16–21]. The COP-NLR before surgery or targeted therapy has also been associated with prognosis in patients with RCC [22, 23]. Additionally, the COP-NLR values are affected by neutropenia and thrombocytopenia as AEs related to sunitinib treatment. In particular, these AEs are more likely to occur within the first 6 weeks of sunitinib treatment.

However, the association between the COP-NLR at the time of AEs during sunitinib treatment and prognosis is unclear, and models combining the prognostic factors of sunitinib have not been well studied. If a prognostic model could be developed, the clinical benefit of continuing sunitinib in the early stage could be determined, leading to the avoidance of serious AEs and longer survival based on long-term treatment with sunitinib. Therefore, we investigated the prognostic factors, including the COP-NLR at the time of AEs within the first 6 weeks of sunitinib treatment, and developed a prognostic model to predict the prognosis of patients with mRCC treated with sunitinib.

## Methods

### Study patients

We performed a retrospective cohort study of 102 patients treated with sunitinib for mRCC at Showa University Hospital, Showa University Northern Yokohama Hospital, and Showa University Fujigaoka Hospital, between June 2008 and August 2020. The data collection limit date was September 30, 2020. All patients were diagnosed with mRCC based on computed tomography (CT) / magnetic resonance imaging (MRI), and, when appropriate, brain imaging, and bone scintigraphy. This study was approved by the Ethics Committee of the Showa University School of Pharmacy.

### Collection of patient data

Patient data and baseline laboratory values were collected from medical records. AEs within the first 6 weeks of sunitinib treatment were collected. To assess early response to treatment, laboratory values within the first 6 weeks of sunitinib treatment were collected.

#### Patient characteristics

The patient background data included sex, age, Eastern Cooperative Oncology Group performance status (PS), histology type, prior nephrectomy, metastatic sites, number of metastatic sites, MSKCC risk groups (favorable-, intermediate-, and poor-risk groups), prior treatment (immunotherapy, targeted therapy), and treatment (first-, second-, and third-line). The drug-related data included the initial dose of sunitinib, treatment schedule, relative dose intensity (RDI) during the first 6 weeks of sunitinib treatment (6-week RDI), and duration of therapy. Blood test data included levels of hemoglobin (Hb), calcium (Ca), aspartate aminotransferase (AST), albumin (Alb), CRP, lactate dehydrogenase (LDH), alkaline phosphatase (ALP), mGPS, neutrophil–lymphocyte ratio (NLR), PLT and COP-NLR.

To investigate prognostic factors, including COP-NLR within the first 6 weeks of sunitinib treatment, we used the MSKCC classification that did not include neutrophil and PLT levels, which are components of COPNLR. COP-NLR within the first 6 weeks of sunitinib treatment is an item reflecting AEs and the early response to sunitinib treatment.

#### Definitions

The MSKCC model was based on five pretreatment variables (Karnofsky PS, LDH concentration, Hb concentration, serum Ca concentration, and time from initial diagnosis to start of systemic treatment) and divided into three risk groups: favorable-risk (0 risk factor), intermediate-risk (1, 2 risk factors), and poor-risk (≥ 3 risk factors) groups. Hypertension was defined as ≥ 140/90 mm Hg. Hypothyroidism was defined as elevated thyroid-stimulating hormone levels with normal triiodothyronine and thyroxine levels. mGPS was defined as follows: patients with elevated CRP levels (> 0.5 mg/dL) and hypoalbuminemia (< 3.5 g/dL) were allocated mGPS 2, patients with only one factor were allocated mGPS 1, and patients with neither factor were allocated mGPS 0. The COP-NLR was defined as follows: patients with elevated platelet levels (> 310 × 10^9^/L) and NLR > 3.5 were allocated COP-NLR 2, patients with only one factor were allocated COP-NLR 1, and patients with neither factor were allocated COP-NLR 0.

#### Division

CRP and Alb levels were divided into two groups according to the lower limit of normal values. AST and ALP levels were divided into two groups according to the upper limit of the normal values. LDH was divided into two groups based on the LDH levels (333 U/L) of the MSKCC model. mGPS and COP-NLR were divided into two groups: moderate (score, 1) or higher.

#### Assessment of response

The response was assessed by CT/MRI performed at 2- to 3- month intervals. Response data presented according to the Response Evaluation Criteria in Solid Tumors (RECIST) v.1.1. were collected from medical records. Progressive disease (PD), which is treatment response data, was collected to calculate PFS.

#### Adverse events

The following AEs related to sunitinib treatment were collected: hypertension, hand-foot syndrome, stomatitis, dysgeusia, oedema, nausea/vomiting, hemorrhage, constipation, diarrhea, fatigue, hypothyroidism, leukopenia, thrombocytopenia, anemia, elevation of AST, elevation of serum creatinine, and elevation of ALP. AEs related to sunitinib treatment were evaluated using the National Cancer Institute Common Terminology Criteria for Adverse Events version 5.0.

### Outcome

The primary outcomes were time to treatment failure (TTF), PFS, and OS. Tumor progression was evaluated based on PD using RECIST.

Time-to-event variables were estimated using the Kaplan–Meier method. TTF was defined as the duration from the first day of sunitinib treatment until the date of discontinuation of sunitinib treatment or death from any cause, whichever came first. PFS was defined as the duration from the first day of sunitinib treatment to the date of tumor progression or death from any cause or the last follow-up visit, whichever came first. OS was defined as the duration from the first day of sunitinib treatment to the date of death from any cause or the last follow-up visit.

### Statistical analysis

#### Baseline and 6-week laboratory value changes

NLR and PLT values at the baseline and within the first 6 weeks of sunitinib treatment were compared by Wilcoxon rank sum tests.

#### The Kaplan–Meier method

Survival curves were estimated using the Kaplan–Meier method. The log-rank test was used to compare survival times between the two groups.

#### Univariate and multivariable analyses

Univariate and multivariable analyses were performed using the Cox proportional hazards model. Significant variables (*p* < 0.05) extracted by univariate analysis were entered into the multivariable analysis. Significant independent variables contributing to the prognosis of patients with mRCC treated with sunitinib were extracted using a stepwise selection method. These data were analyzed by using the SPSS software, version 27 (IBM, Tokyo, Japan). Statistical significance was set at *P* < 0.05.

#### Prognostic model and assessment

##### Prognostic model

Each prognostic model was developed using prognostic factors extracted by multivariable analysis. The β values for these factors were derived from the smallest β value among the prognostic factors, approximated to the nearest integer. For each factor, the approximate β values were scored as integers. For each patient, the scores were calculated as the sum of the scores for each factor. Patients were divided into three groups (low-, intermediate-, and high-risk) based on the distribution of their scores. Survival curves of the three groups were estimated using the Kaplan–Meier method. The log-rank test was used to compare survival times among the three groups in prognostic models for TTF, PFS, and OS.

##### Prognostic nomogram

A nomogram for possible prognostic factors was formulated to provide visualized risk prediction using R software with the rms package. A nomogram was established through Cox regression model analysis according to prognostic factors of OS (i.e., the number of metastatic sites, MSKCC risk group, non-hypertension, mGPS, and 6-week COP-NLR).

##### Assessment

Calibration of the prognostic model and nomogram for OS was performed by comparing the predicted outcomes with the observed outcomes. The performance of the prognostic model and nomogram for predicting survival was evaluated with Harrell’s concordance index (c-index) which is a measure of discrimination. The maximum value of the c-index is 1.0, which indicates perfect discrimination. The c-index of 0.5 indicates a random chance to correctly discriminate the outcome.

## Results

### Patient characteristics

The characteristics of the 102 patients are shown in Table 1. The median age was 67.5 (range, 28–83) years, and 83 (81.4%) were males. The MSKCC risk groups were favorable in 13 patients (12.8%), intermediate in 70 (68.6%), and poor in 19 (18.6%). The median follow-up period was 23.6 (range, 0.2–135.3) months. The median duration of sunitinib treatment was 4.7 (range, 0.2–67.1) months. Seventy-five patients (73.5%) received sunitinib for at least 6 weeks.

**Table 1:** Patient characteristics

Characteristics (*n* = 102)	N	%	Characteristics	N	%
**Patient characteristics**			AST		
Sex			≤ 30 U/L	83	81.4
Male	83	81.4	> 30 U/L	19	18.6
Age			Alb		
Median (range), years	67.5 (28–83)		< 3.5 g/dL	33	32.4
ECOG-PS			≥ 3.5 g/dL	69	67.6
0	64	62.7	CRP		
1	23	22.6	≤ 0.5 g/dL	44	43.1
≥ 2	15	14.7	> 0.5 g/dL	58	56.9
Histology type			ALP		
Clear cell	76	96.2	≤ 322 U/L	61	61.6
Non-clear cell	3	3.8	> 322 U/L	38	38.4
Prior nephrectomy	75	73.5	mGPS		
Metastatic sites			0	42	41.2
Lung	69	67.6	1	28	27.5
Bone	25	24.5	2	32	31.3
Lymph node	30	29.4	NLR		
Liver	15	14.7	≤ 3.5	64	64
Other	23	22.5	> 3.5	36	36
Number of metastatic sites			PLT		
0	3	2.9	≤ 310 × 10^9^/L	71	69.6
1	45	44.2	> 310 × 10^9^/L	31	30.4
≥ 2	54	52.9	COP-NLR		
MSKCC risk group			0	49	49
Favorable	13	12.8	1	36	36
intermediate	70	68.6	2	15	15
poor	19	18.6	**Laboratory data within the first 6 weeks of sunitinib**		
**Treatment characteristics**			Hb		
Prior immunotherapy	21	20.6	< 12 g/dL	55	53.9
Prior targeted therapy	13	12.7	≥ 12 g/dL	47	46.1
Treatment			Ca		
1st line	75	73.5	< 10 g/dL	80	83.3
2st line	18	17.7	≥ 10 g/dL	16	16.7
3st line	9	8.8	LDH		
Initial dose			≤ 333 U/L	76	76
50 mg	47	46	> 333 U/L	24	24
37.5 mg	42	41.2	AST		
25 mg	12	11.8	≤ 30 U/L	62	60.8
12.5 mg	1	1	> 30 U/L	40	39.2
Treatment schedule			CRP		
4-week on / 2-week off	35	34.3	≤ 0.5 g/dL	26	25.7
2-week on / 1-week off	64	62.7	> 0.5 g/dL	75	74.3
Other	3	3	mGPS		
**RDI during the first 6 weeks of sunitinib**			0	24	24.2
Median (range), %	62.5 (17.9–100)		1	26	26.3
Duration of therapy			2	49	49.5
Median (range), days	140 (5–2012)		NLR		
**Laboratory data at start of sunitinib**			≤ 3.5	71	74
Hb			> 3.5	25	26
< 12 g/dL	55	53.9	PLT		
≥ 12 g/dL	47	46.1	≤ 310×10^9^/L	97	95.1
Ca			> 310×10^9^/L	5	4.9
< 10 g/dL	82	83.7	COP-NLR		
≥ 10 g/dL	16	16.3	0	71	74
LDH			1	22	22.9
≤ 333 U/L	91	90.1	2	3	3.1
> 333 U/L	10	9.9			

*ECOG PS* Eastern Cooperative Oncology Group Performance Status, *MSKCC* Memorial Sloan Kettering Cancer Center, *RDI* Relative Dose Intensity, *Hb* Hemoglobin, *Ca* Calcium, *LDH* Lactate dehydrogenase, *AST* Aspartate aminotransferase, *Alb* Albumin, *CRP* C-reactive protein, *ALP* Alkaline phosphatase, *mGPS* Modified Glasgow Prognostic Score, *NLR* Neutrophil–lymphocyte ratio, *PLT* Platelet count, *COP-NLR* Combination of platelet count and neutrophil–lymphocyte ratio

NLR and PLT levels within the first 6 weeks of sunitinib treatment were significantly reduced compared with those at baseline (median NLR; 1.9 [range, 0.4–48.5] vs. 2.9 [range, 0.9–23.0], *p* < 0.001, median PLT (10^9^/L); 123 [range, 22–434] vs. 244 [range, 94–786], *p* < 0.001).

### Outcome

**Fig. 1:**
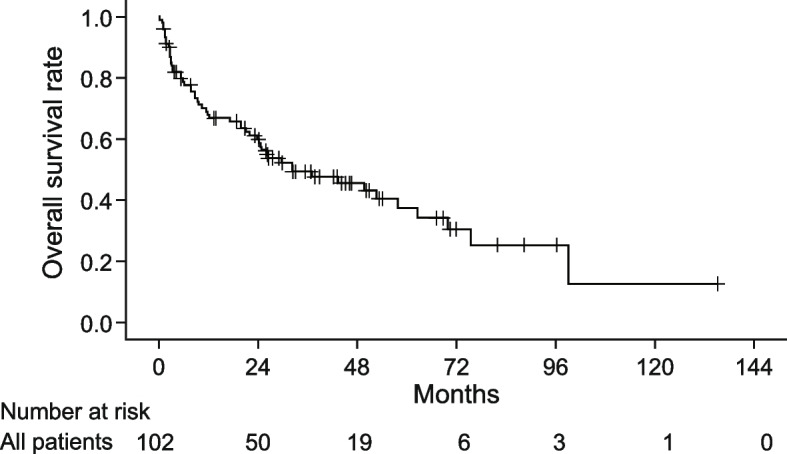
Cumulative survival curve of all patients

The cumulative survival curve for all patients is shown in Fig. 1. The median TTF, PFS, and OS were 4.9, 5.8, and 32.3 months, respectively. During the follow-up period, 17 patients (16.7%) discontinued sunitinib due to AEs, 87 patients (85.3%) experienced disease progression, and 55 patients (53.9%) died of any cause.

### Univariate and multivariable analyses

The results of univariate and multivariable analyses are summarized in Table 2. Among the factors that were significant in the univariate analysis, multivariable analysis was performed, except for those that were correlated. In the multivariable analysis, the number of metastatic sites, AST, ALP, 6-week RDI, and 6-week COP-NLR were significantly associated with TTF. Additionally, the number of metastatic sites, MSKCC risk group, non-hand-foot syndrome, and 6-week COP-NLR were significantly associated with PFS. Moreover, the number of metastatic sites, MSKCC risk group, non-hypertension, mGPS, and 6-week COP-NLR were significantly associated with OS.

**Table 2:** Univariate and multivariable analyses of factors associated with time to treatment failure, progression-free-survival, and overall survival

Variables	Time to treatment failure				Progression-free-survival				Overall survival			
	Univariate		Multivariable^b^		Univariate		Multivariable^c^		Univariate		Multivariable^d^	
	HR	*P*	HR	*P*	HR	*P*	HR	*P*	HR	*P*	HR	*P*
			(β)				(β)				(β)	
**Characteristics**												
Sex (female vs. male)	0.987	0.960			1.115	0.682			0.890	0.734		
Age (≥ 65 vs. < 65 years)	1.624	0.024			1.256	0.290			0.822	0.474		
ECOG PS (≥ 2 vs. 0, 1)	1.520	0.165			1.452	0.216			2.599	0.012		
MSKCC risk group (poor vs. others)	2.934	< 0.001			3.340	< 0.001	3.234	< 0.001	9.115	< 0.001	7.239	< 0.001
							(1.174)				(1.979)	
Prior nephrectomy (yes vs. no)	0.651	0.071			0.650	0.068			0.308	< 0.001		
Number of metastatic sites (≥ 2 vs. 0, 1)	1.960	0.002	2.322	< 0.001	2.369	< 0.001	2.273	< 0.001	2.756	0.001	3.260	< 0.001
			(0.842)				(0.821)				(1.182)	
Treatment (2, 3 vs. 1st line)	1.023	0.921			1.188	0.455			1.131	0.687		
6-week RDI (< 60 vs. ≥ 60%)	2.671	< 0.001	3.160	< 0.001	1.321	0.190			1.581	0.095		
			(1.151)									
Duration of therapy (< 6 vs. ≥ 6 weeks)	―	―			2.375	< 0.001			2.088	0.014		
**Adverse events**^a^												
Hypertension (no vs. yes)	1.371	0.136			1.194	0.412			2.169	0.005	2.073	0.024
											(0.729)	
Hand-foot syndrome (no vs. yes)	1.537	0.046			1.529	0.049	1.664	0.043	1.203	0.499		
							(0.509)					
Diarrhea (no vs. yes)	0.668	0.080			0.673	0.087			0.563	0.047	1.903	0.066
											(0.643)	
Hypothyroidism (no vs. yes)	1.495	0.099			1.414	0.154			1.085	0.790		
Leukopenia (no vs. yes)	0.832	0.389			0.650	0.047			0.704	0.206		
Thrombocytopenia (no vs. yes)	1.263	0.350			0.942	0.814			1.649	0.112		
Elevation of ALP (no vs. yes)	0.740	0.159			0.695	0.093			0.457	0.007		
**Laboratory data at start of sunitinib**												
Hb (≥ 12 vs. < 12 g/dL)	0.504	0.001			0.514	0.002			0.280	< 0.001		
Ca (≥ 10 vs. < 10 g/dL)	1.841	0.029			1.987	0.017			4.009	< 0.001		
LDH (> 333 vs. ≤ 333 U/L)	5.395	< 0.001			7.278	< 0.001			14.550	< 0.001		
AST (> 30 vs. ≤ 30 U/L)	2.160	0.003	2.303	0.009	2.060	0.006			2.689	0.002		
			(0.834)									
Alb (≥ 3.5 vs. < 3.5 g/dL)	0.550	0.008			0.469	0.001			0.225	< 0.001		
CRP (> 0.5 vs. ≤ 0.5 g/dL)	2.239	< 0.001			2.055	0.001			4.465	< 0.001		
ALP (> 322 vs. ≤ 322 U/L)	2.102	< 0.001	1.811	0.013	2.253	< 0.001			3.134	< 0.001		
			(0.594)									
mGPS (1, 2 vs. 0)	2.037	0.001			2.134	< 0.001			5.119	< 0.001	2.946	0.005
											(1.080)	
NLR (> 3.5 vs. ≤ 3.5)	1.319	0.207			1.519	0.059			3.080	< 0.001		
PLT (> 310 vs. ≤ 310 × 10^9^ /L)	1.318	0.223			1.457	0.098			2.290	0.005		
COP-NLR (1, 2 vs. 0)	1.271	0.256			1.327	0.184			2.823	< 0.001		
**Laboratory data within the first 6 weeks of sunitinib**												
Hb (≥ 12 vs. < 12 g/dL)	0.611	0.020			0.507	0.002			0.270	< 0.001		
Ca (≥ 10 vs. < 10 g/dL)	3.798	< 0.001			3.639	< 0.001			5.053	< 0.001		
LDH (> 333 vs. ≤ 333 U/L)	2.218	0.001			2.397	< 0.001			3.531	< 0.001		
AST (> 30 vs. ≤ 30 U/L)	1.311	0.202			1.414	0.107			1.185	0.536		
Alb (≥ 3.5 vs. < 3.5 g/dL)	0.527	0.003			0.510	0.002			0.396	0.001		
CRP (> 0.5 vs. ≤ 0.5 g/dL)	1.732	0.026			1.718	0.034			1.421	0.286		
mGPS (1, 2 vs. 0)	1.749	0.026			1.765	0.030			1.348	0.366		
NLR (> 3.5 vs. ≤ 3.5)	2.898	< 0.001			2.799	< 0.001			4.357	< 0.001		
PLT (> 310 vs. ≤ 310 × 10^9^ /L)	5.578	< 0.001			5.142	< 0.001			7.758	< 0.001		
COP-NLR (1, 2 vs. 0)	2.898	< 0.001	2.255	0.003	2.799	< 0.001	2.270	0.004	4.357	< 0.001	2.860	0.002
			(0.813)				(0.820)				(1.051)	

^a^Adverse events were developed within the first 6 weeks of sunitinib. *HR* Hazard ratio, *β* Beta-coefficient, *ECOG PS* Eastern Cooperative Oncology Group Performance Status, *MSKCC* Memorial Sloan Kettering Cancer Center, *RDI* Relative Dose Intensity, *Hb* Hemoglobin, *Ca* Calcium, *LDH* Lactate dehydrogenase, *AST* Aspartate aminotransferase, *Alb* Albumin, *CRP* C-reactive protein, *ALP* Alkaline phosphatase, *mGPS* Modified Glasgow Prognostic Score, *NLR* Neutrophil–lymphocyte ratio, *PLT* Platelet count, *COP-NLR* Combination of platelet count and neutrophil– lymphocyte ratio

^b^Age, MSKCC risk group, number of metastatic sites, hand-foot syndrome, 6-week RDI, AST, ALP, mGPS, 6-week mGPS, and 6-week COP-NLR were subjected to multivariate analysis

^c^MSKCC risk group, number of metastatic sites, duration of therapy, hand-foot syndrome, leukopenia, mGPS, AST, ALP, 6-week mGPS, and 6-week COP-NLR were subjected to multivariate analysis

^d^MSKCC risk group, prior nephrectomy, number of metastatic sites, duration of therapy, hypertension, diarrhea, elevation of ALP, AST, ALP, mGPS, COP-NLR, 6-week Alb, and 6-week COP-NLR were subjected to multivariate analysis

### Survival curves according to the 6-week COP-NLR

**Fig. 2:**
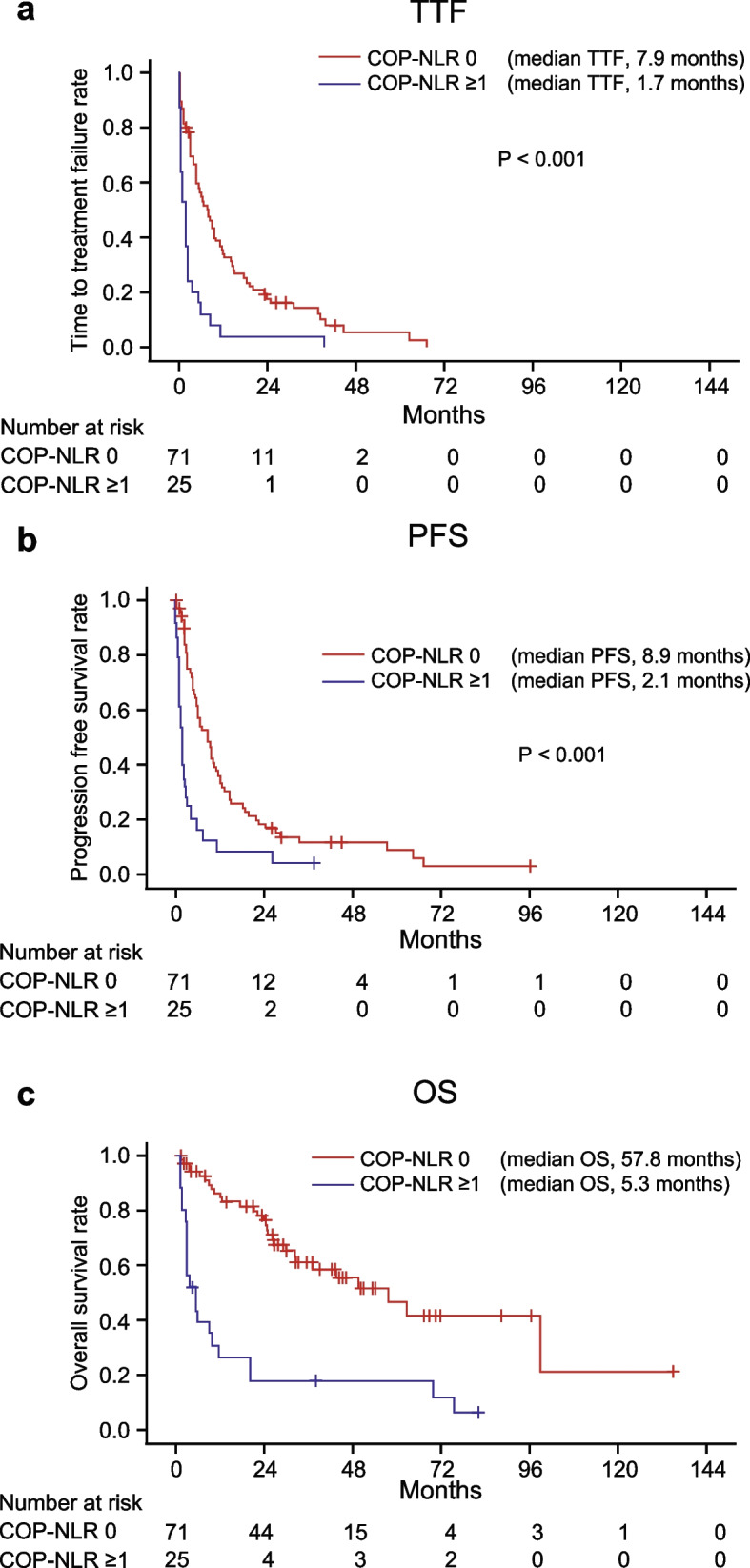
Kaplan–Meier curves of (**a**) time to treatment failure, (**b**) progression-free-survival, and (**c**) overall survival in the two groups divided according to the 6-week COP-NLR

The Kaplan–Meier curves of TTF, PFS, and OS according to the 6-week COP-NLR are shown in Fig. 2. A higher 6-week COP-NLR was significantly associated with shorter TTF, PFS, and OS (*p* < 0.001).

### Prognostic model and assessment

#### Prognostic model

The integer scores assigned from the β value of prognostic factors for TTF were as follows: 1 point for the number of metastatic sites, AST, ALP, and 6-week COP-NLR; and 2 points for 6-week RDI. The sum of the scores of the five factors, ranging from 0 to 6, was calculated for all patients. The patients were divided into three groups: low-risk group (≤ 1 point; *n* = 37), intermediate-risk group (2–3 points; *n* = 36), and high-risk group (≥ 4 points; *n* = 20). Additionally, the integer scores assigned from the β value of prognostic factors for PFS were as follows: 1 point for non-hand-foot syndrome; and 2 points for the number of metastatic sites, MSKCC risk group, and 6-week COP-NLR. The sum of the scores of the five factors, ranging from 0 to 7, was calculated for all patients. The patients were divided into three groups: low-risk group (≤ 1 point; *n* = 34), intermediate-risk group (2–3 points; *n* = 36), and high-risk group (≥ 4 points; *n* = 26). Moreover, the integer scores assigned from the β value of prognostic factors for OS were as follows: 1 point for non-hypertension, mGPS, and 6-week COP-NLR; 2 points for the number of metastatic sites; and 3 points for the MSKCC risk group. The sum of the scores of the five factors, ranging from 0 to 8, was calculated for all patients. The patients were divided into three groups: low-risk group (≤ 1 point; *n* = 30), intermediate-risk group (2–3 points; *n* = 32), and high-risk group (≥ 4 points; *n* = 34).

**Fig. 3:**
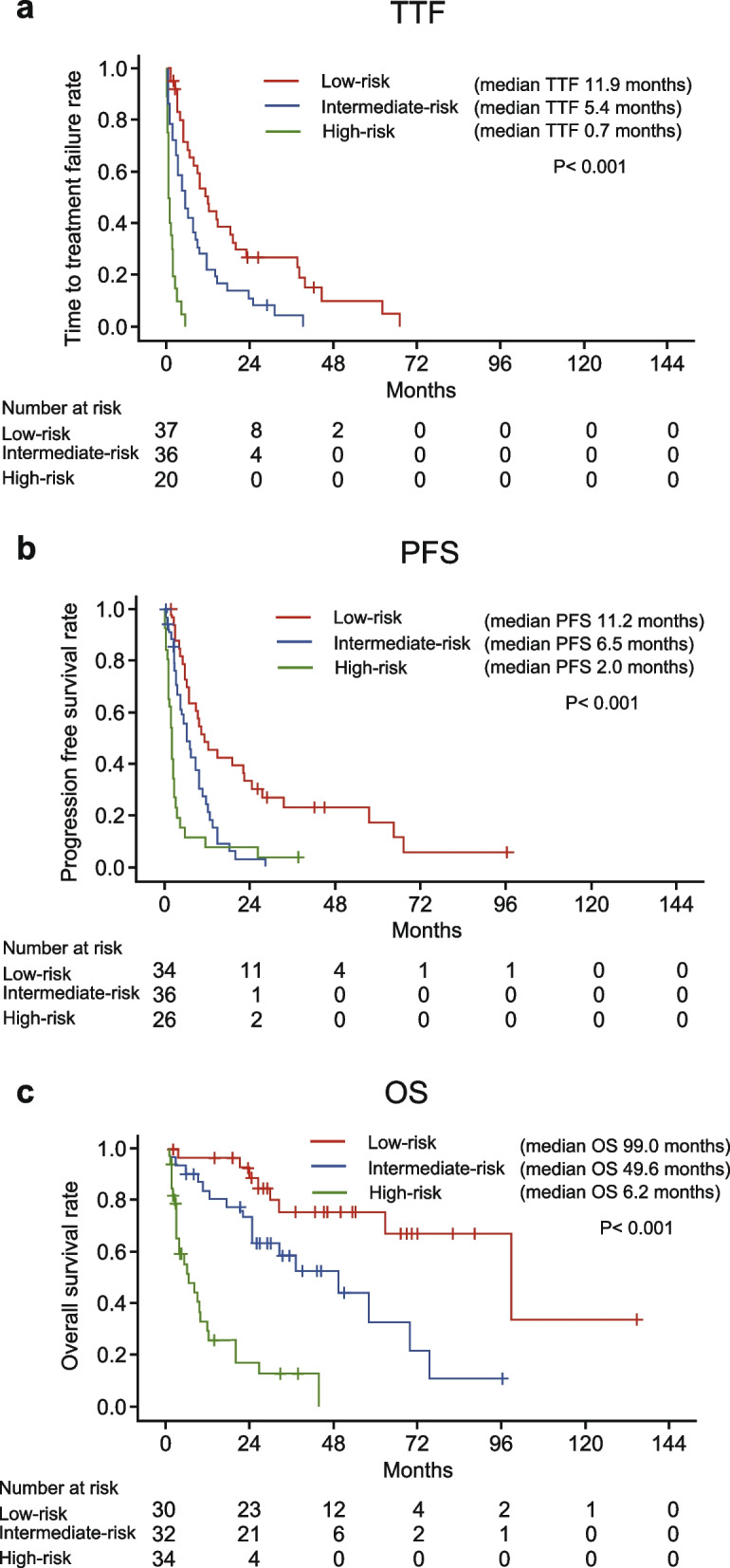
Kaplan–Meier curves of (**a**) time to treatment failure, (**b**) progression-free-survival, and (**c**) overall survival in the three groups divided according to the prognostic model

The Kaplan–Meier curves of TTF, PFS, and OS according to the prognostic models are shown in Fig. 3. There were significant differences among the three groups in the prognostic models for TTF, PFS, and OS (*p* < 0.001). For internal validation, the bootstrapped calibration plot of the model predicting 1-year OS performed well with the ideal model (Supplemental Fig. 1). The C-index of model was 0.757 (95% confidence interval [CI]: 0.699–0.816).

#### Prognostic nomogram

Each factor in the nomogram was assigned a weighted number of points, and the sum of points for each patient was in accordance with a specific predicted 1- and 3-year OS (Supplemental Fig. 2). For internal validation, the bootstrapped calibration plot of the nomogram predicting 1- and 3-year OS performed well with the ideal model (Supplemental Fig. 3). The C-index of nomogram was 0.821 (95% CI: 0.762–0.880).

### Adverse events

The most common treatment-related AEs associated with sunitinib are shown in Table 3. The most common AEs of all grades were hypertension in 60 patients (58.8%), hand-foot syndrome in 49 (48.0%), leukopenia in 55 (53.9%), and thrombocytopenia in 79 (77.5%). The most common grade 3/4 AEs were hypertension in 20 patients (19.6%), hand-foot syndrome in 7 (6.9%), leukopenia in 10 (9.8%), and thrombocytopenia in 17 (16.7%).

**Table 3:** Treatment-related adverse events

Adverse events	All grades	(n, %)	Grade3-4	(n, %)
Hypertension	60	(58.8)	20	(19.6)
Hand-foot syndrome	49	(48.0)	7	(6.9)
Stomatitis	36	(35.3)	6	(5.9)
Dysgeusia	17	(16.7)	-	-
Oedema	14	(13.7)	0	(0)
Nausea / Vomiting	24	(23.5)	0	(0)
Hemorrhage	26	(25.5)	0	(0)
Constipation	16	(15.7)	0	(0)
Diarrhea	29	(28.4)	3	(2.9)
Fatigue	46	(45.1)	7	(6.9)
Hypothyroidism	26	(25.5)	5	(4.9)
Leukopenia	55	(53.9)	10	(9.8)
Thrombocytopenia	79	(77.5)	17	(16.7)
Anemia	70	(68.6)	5	(4.9)
Elevation of AST	63	(61.8)	8	(7.8)
Elevation of serum creatinine	53	(52.0)	5	(4.9)
Elevation of ALP	51	(51.5)	1	(1.0)

*AST* Aspartate aminotransferase, *ALP* Alkaline phosphatase

## Discussion

In this study, we first demonstrated that the COP-NLR within the first 6 weeks of sunitinib treatment had a greater impact on OS than the COP-NLR at the start of sunitinib treatment. The 6-week COP-NLR, which reflects the early response to sunitinib treatment and bone marrow suppression, may be a useful prognostic indicator of the benefit from sunitinib. To the best of our knowledge, this is the first study to reveal the relationship between prognosis and the 6-week COP-NLR. Prognostic factors reflecting the early response to sunitinib treatment, such as the 6-week COP-NLR, have not been previously reported. Therefore, this study had a high clinical application value. Moreover, the developed prognostic model for OS with the addition of 6-week COP-NLR to the existing prognostic factors accurately predicted the prognosis of patients with mRCC treated with sunitinib. Thus, this model may provide clinical criteria for the continuation of sunitinib treatment in the early stages of mRCC.

The higher 6-week COP-NLR indicated that sunitinib did not reduce the number of platelets and neutrophils in the blood. Sunitinib exhibits a dose- and time-dependent antitumor effect [24]. In the absence of the occurrence of thrombocytopenia, the antitumor effect of vascular endothelial growth factor receptor (VEGFR) inhibition is not achieved and may lead to a shorter OS. In tumor progression, neutrophils and lymphocytes, which are components of the COP-NLR, are associated with the tumor microenvironment. Neutrophils are involved in tumor progression, and lymphocytes play a role in antitumor immunity [25]. Platelets induce epithelial-to-mesenchymal transition in cancer and promote metastasis from the primary site [26]. Angiogenic factors and growth factors released from platelets promote tumor angiogenesis, tumor growth, and metastasis [27]. Therefore, in the absence of neutropenia or thrombocytopenia, cytokines released from neutrophils may cause tumor growth and progression. NLR and PLT levels within the first 6 weeks of sunitinib treatment were significantly reduced compared to those at baseline. This suggests that the early response of NLR and PLT levels was associated with improved prognosis. Therefore, the 6-week COP-NLR is a useful prognostic factor combined with bone marrow suppression and early response to sunitinib treatment.

In addition to the 6-week COP-NLR, the MSKCC risk group, number of metastases, non-hypertension, and mGPS were significantly associated with OS. These prognostic factors were similar to those reported previously [6, 8] Additionally, sunitinib-induced hypertension is correlated with the effects of VEGFR inhibition [12]. In the absence of the occurrence of hypertension, the effect of VEGFR inhibition is not achieved and may lead to a shorter OS. Non-hypertension is an important prognostic indicator because it has been previously reported as a prognostic factor [13].

We showed that the developed OS prognostic model accurately predicted the prognosis of patients with mRCC treated with sunitinib. In this developed prognostic model, integrating the 6-week COP-NLR into the existing prognostic factors may improve discrimination between groups and thus improve individual risk prediction. Although COP-NLR has previously been shown to be useful at baseline prior to TKI [22], this study is the first to show that alterations of these values may provide additional prognostic information.

In this study, a prognostic nomogram and model for OS were developed; the C-index of the nomogram was higher than that of the model. However, the advantage of the developed prognostic model is that it is easy to stratify prognostic risk into three groups based on simple scores. The model is simple and easy to use in clinical practice, making it a useful tool to assist providers in determining appropriate treatment according to their prognostic risk for patients with mRCC. Therefore, the model is useful tool for decision-making to continue sunitinib in the early treatment stage for patients with mRCC. The low-risk group achieved an antitumor effect from the VEGFR inhibitor sunitinib, which is expected to lead to a longer OS. On the other hand, in the high-risk group, a longer OS cannot be expected even if sunitinib is selected, so it is necessary to consider changing to other molecular-targeted agents or immune checkpoint inhibitors.

In this study, we also investigated the impact of TTF and PFS on prognosis. In mRCC, it is important to use a highly effective drug at an early stage for as long as possible as the effect of tumor burden reduction in the early stage of sunitinib treatment affects subsequent prognosis [5]. Additionally, it has been reported that long-term treatment at a dose to achieve tumor burden reduction is associated with a favorable prognosis. Therefore, PFS associated with tumor growth and TTF associated with treatment continuation are considered to have a strong impact on the prognosis of mRCC. The prognostic factors of TTF and PFS may be important indicators for selecting a targeted agent for mRCC.

Non-hand-foot syndrome, high AST (> 30 U/L), ALP (> 322 U/L) levels, and 6-week RDI (< 60%) were extracted as prognostic factors for PFS and TTF, respectively. Hand-foot syndrome is a favorable prognostic factor for sunitinib [28]. High AST and ALP levels are associated with liver and bone metastases and indicate poor PS [7, 29]. The high AST group had liver metastases in 26.3% of cases, and the high ALP group had bone metastases in 31.6% of cases (data not shown). Additionally, because sunitinib is metabolized in the liver, early liver toxicity is likely to lead to discontinuation of sunitinib at the early stage.

## Limitations

The present study has two limitations. First, there were few patients treated with sunitinib as first-line therapy; therefore, a prognostic model could not be developed for patients with mRCC treated with sunitinib as first-line therapy. Second, a prognostic model that included the severity of AEs could not be developed.

## Conclusions

This study showed that the COP-NLR within the first 6 weeks of sunitinib treatment had a greater impact on OS than the COP-NLR at the start of sunitinib treatment. We showed that the developed prognostic model for OS with the addition of 6-week COP-NLR to the prognostic factors at baseline accurately predicted the prognosis of patients with mRCC treated with sunitinib. The developed prognostic model for OS, including the 6-week COP-NLR, will be useful in decision-making to continue sunitinib in the early treatment stage of patients with mRCC. The low-risk group can achieve the antitumor effect of the VEGFR inhibitor sunitinib, which is expected to lead to longer OS.

## Supplementary Information

**Additional file 1: Supplemental Fig. 1.** Calibration curves for predicting 1-year overall survival using prognostic model. Prognostic model-predicted 1- year overall survival is plotted on the x-axis, and actual overall survival is plotted on the y-axis. The dotted line represents an ideal prognostic model, and the solid line represents the current prognostic model. The vertical bars are 95% confidence intervals, and the ×'s are bootstrap-corrected estimates.

**Additional file 2: Supplemental Fig. 2.** A nomogram for 1- and 3-year overall survival for patients treated with sunitinib for mRCC.

**Additional file 3: Supplemental Fig. 3.** Calibration curves for predicting (a) 1-year overall survival using nomogram and (b) 3- year OS using nomogram. Nomogram-predicted 1- and 3-year overall survival are plotted on the x-axis, and actual 1- and 3-year overall survival are plotted on the y-axis. The dotted line represents an ideal nomogram, and the solid line represents the current nomogram. The vertical bars are 95% confidence intervals, and the ×'s are bootstrap-corrected estimates.

## Data Availability

The datasets generated during and analyzed during the current study are not publicly available due to ethical reasons but are available from the corresponding author on reasonable request.

## Abbreviations

AEs: Adverse events
Alb: Albumin
ALP: Alkaline phosphatase
AST: Aminotransferase
Ca: Calcium
CI: Confidence interval
COP-NLR: Combination of platelet count and neutrophil–lymphocyte ratio
CRP: C-reactive protein
CT: Computed tomography
Hb: Hemoglobin
LDH: Lactate dehydrogenase
mGPS: Modified glasgow prognostic score
mRCC: Metastatic renal cell carcinoma
MRI: Magnetic resonance imaging
MSKCC: Memorial sloan kettering cancer center
NLR: Neutrophil–lymphocyte ratio
OS: Overall survival
PD: Progressive disease
PFS: Progression-free survival
PLT: Platelet count
PS: Performance status
RCC: Renal cell carcinoma
RDI: Relative dose intensity
RECIST: Response evaluation criteria in solid tumors
TTF: Time to treatment failure
VEGFR: Vascular endothelial growth factor receptor
